# Impact of Natural Phytosanitary Product Residuals on Yeast Fermentation Performance and Wine Composition

**DOI:** 10.3390/foods13213484

**Published:** 2024-10-30

**Authors:** Natascia Bartolozzi, Francesco Maioli, Monica Picchi, Valentina Civa, Valentina Canuti, Paola Domizio

**Affiliations:** Department of Agricultural, Food, Environmental, and Forestry Sciences and Technologies (DAGRI), University of Florence, Piazzale delle Cascine 18, 50144 Firenze, Italy; natascia.bartolozzi@unifi.it (N.B.); francesco.maioli@unifi.it (F.M.); monica.picchi@unifi.it (M.P.); valentina.civa@unifi.it (V.C.); paola.domizio@unifi.it (P.D.)

**Keywords:** pesticide, alcoholic fermentation, wine quality, *Botrytis cinerea*, fermentation kinetics, new fungicides, essential oils, organic wine

## Abstract

Although phytosanitary treatments are necessary to protect grapes from parasitic diseases, consumers are increasingly concerned about the use of synthetic phytosanitary products and their possible residues in wine. Pre-harvest phytosanitary treatments are often inevitable, and consequently downtime is required to avoid possible residues on the grapes. Instead, natural phytosanitary products, such as essential oil (EO)-based products, can be applied close to the harvest without specific restrictions, with results that are not only technically convenient but also more attractive for the consumers. Because of the high antimicrobial activity of EO products, in the present study we evaluated the effect of different residual amounts of two new EO-based phytosanitary products on the alcoholic fermentation and the chemical composition of the final fermented products. In particular, two EO-based new formulations, exploitable in organic viticulture management, were evaluated. Increasing concentrations of each formulation were tested during laboratory scale fermentations and in comparison with synthetic and natural commercial phytosanitary products. Growth and fermentation kinetics of a commercial yeast strain of *Saccharomyces cerevisiae* and the chemical and sensory profiles of the final products were evaluated. Both new formulations showed no significant impact on the growth and fermentation kinetic of *S. cerevisiae* at any of the concentrations tested. In all trials, alcoholic fermentation was completed in 15 days. Instead, a different chemical composition of the final products was observed. Therefore, these new products might represent an interesting alternative tool to the conventional phytosanitary treatments, being applicable close to the harvest without negative impacts on the kinetics of alcoholic fermentation and also being more acceptable to wine consumers.

## 1. Introduction

Organic farming is a growing sector in all world markets due to a more widespread awareness of how crucial it is to pay more attention to the environment in the agri-food sector. The choice of increasingly environmentally friendly solutions is essential to meet the demand for health-awareness on the part of consumers who turn their preferences towards products with traceability and minimum impact. In the wine sector, many producers have chosen to manage their vineyards following the organic method, and every year new adhesions and consensuses can be counted. In the viticultural field, a major challenge is the availability of plant protection products that are allowed in the organic field [1,2,3,4,5].

Phytosanitary products conventionally used in vineyard treatments are synthetic antifungals, or copper in organic management, which are necessary to control diseases on the green parts of the vine and on grapes. In viticulture, preventive spraying programs are applied for disease control of powdery mildew (*Erysiphe necator*), downy mildew (*Plasmopara viticola*), and gray mold (*Botrytis cinerea*). They are among the most damaging diseases for cultivated grapes (*Vitis vinifera*) worldwide, leading to severe lesions and resulting in significant commercial losses [6,7]. *Botrytis* occurs in pre-harvest and can cause severe damage to grape quality and great losses. The defense against this pathogen is made even more complex by the timeline when it occurs, i.e., close to harvesting [8]. The problem, due to pre-harvest phytosanitary treatments, is caused by possible residues on the grapes and the direct consequences on the quality of the wines; therefore, downtime after pesticide application is required.

The amount of residual phytosanitary products is estimated in a range of 0.003–38 mg/kg in grapes, 0.0038–34.52 mg/kg in musts, and 0.00015–5.99 mg/kg in wines [9]. A considerable number of practices emerge each year that seek to mitigate these issues, including integrated pest management programs, which aim to minimize the health and environmental impacts of phytosanitary products [10], as well as imposing pre-harvest safety limits [11]. Although these actions contribute to reducing the quantity of agrochemicals used and therefore the health risks for consumers, they do not guarantee the absence of chemical residues on grapes and do not consider their possible effects on further industrial processing.

For the purposes of assessing the dissipation kinetics of phytosanitary product residues during winemaking, some authors [12] evaluated five fungicides commonly used for plant and bunch defense against powdery mildew (boscalid, kresoxim-methyl, metrafenone) and gray mold (mepanipyrim, fenhexamid). In all cases the fungicides dissipated by more than 68%, confirming the detoxifying effect of the various winemaking procedures. Several authors [9,13,14,15,16,17,18] investigated the effects of synthetic fungicide residuals on grapes during alcoholic fermentation. Results highlighted that synthetic compounds could have a negative impact on yeast activity affecting the fermentation performance. Consequently, the biosynthesis of volatile compounds and their concentration on the final wine may be subject to alterations [19]. Phytosanitary treatments can influence not only *S. cerevisiae* performance, but also the indigenous yeasts on grapes’ skin, thus reducing microbial biodiversity. It is worth highlighting here also that high residues of copper, used in organic management of vineyards, can be detrimental to the winemaking process and wine quality [20,21,22]. Moreover, the history of the development of fungicide resistance in plant pathogenic fungi reveals that *B. cinerea* (especially on grapevines) shows an elevated risk of this phenomenon. From 2003 to 2017, more than 5000 isolates of *B. cinerea* were collected in China from cultures and have been used to verify resistance to those fungicides commonly applied [23]. The results of this study showed that *B. cinerea* increased the resistance to MBCs (methyl-benzimidazole carbamates), DCFs (dicarboximides), APs (anilinopyrimidines), QoIs (quinone outside inhibitors), SDHIs (succinate dehydrogenase inhibitors) and PPs (phenylpyrroles). Due to the ability of *B. cinerea* to rapidly develop resistance, several strategies have been developed over the last decade to manage this issue, including with the development of bio-agrochemicals.

Essential oils (EOs), terpenoids, saponins, phenolic compounds, alkaloids, peptides, and proteins have been shown to have high antiviral, antiparasitic, insecticidal and antifungal activity [8,24,25,26,27]. EOs are aromatic oily liquids obtained from plant material (flowers, buds, seeds, leaves, twigs, bark, herbs, wood, fruits, and roots) and are secondary metabolites of plants. They are synthesized either in the cytoplasm or in chloroplasts. The main chemical classes of EOs are terpenes and terpenoids, extracted by steam distillation [28]. The advantages of EOs include low toxicity to humans and non-toxic-organism targeting, broad-spectrum efficacy, and multiple mechanisms of action.

Minimum Inhibitory Concentration (MIC) is generally used to measure the antibacterial performance of EOs. Generally, EOs containing a high proportion of volatiles, such as carvacrol, eugenol, and thymol, have the strongest antibacterial properties against foodborne pathogens. Several EOs, or their components, are registered as flavorings in food products by the European Commission.

As far as is known, no specific studies have been carried over to evaluate the impact of EOs as phytosanitary products on the yeast fermentation performance and on the quality of the fermented products. Hence, the aim of the present study was to evaluate the impact of two new natural phytosanitary products, based on EO mixtures obtained from Apiaceae, Lamiaceae, and Geraniaceae family plants, on *S. cerevisiae* performance during alcoholic fermentation carried out in synthetic grape juice. Two commercial phytosanitary products (natural and synthetic) were used for comparison determination. Fermented products were also evaluated to highlight possible differences in their final composition (i.e., volatiles).

## 2. Materials and Methods

### 2.1. Phytosanitary Products and Theoretical Vineyard Treatments

Two new EO-based formulations (P1 and P2), as alternative phytosanitary products for vineyard treatments, were supplied by the Institute for Environmental Solutions, Cesu parish, Latvia. P1 formulation is based on a mixture of EOs of Apiaceae and Lamiaceae family plants, containing 1,8-cineole 0.1%, a-phellandrene 0.6%, β-caryophyllene 0.4%, β-pinene 0.2%, carvacrol 2.2%, carvone 4.9%, eugenol 6.0%, limonene 5.1%, p-cymene 0.1%, and γ-terpinene 0.3%, while P2 is based on a mixture of EOs of Lamiaceae and Geraniaceae family plants, containing α-pinene 0.7%, β-caryophyllene 0.8%, eugenol 2.3%, geraniol 4.3%, limonene 1.2%, linalool 4.4%, thymol 5.0%, p-cymene 0.2%, and γ-terpinene 0.5%. The two new products were evaluated in comparison with two commercially available phytosanitary products, a synthetic one (P3, “Switch”, Syngenta Group Co., Basilea, Swiss; cyprodinil 37.5% and fludioxonil 25%) and a natural one (P4, 3Logy, Sipcam Italia spa, Milano, Italy; eugenol 3.2%, geraniol 6.4%, and thymol 6.4%) (Table 1).

In order to estimate the phytosanitary residue of each product (P1–P4) on the synthetic grape juice, a theoretical vineyard treatment was calculated according to the following model: a grape production of 100 quintals in the area of 1 hectare, and 100 L of water for each phytosanitary treatment dosage [29]. The phytosanitary residues on the synthetic grape juice were intentionally overestimated. Drift loss, evaporation, and ground loss during the vineyard treatment were not considered [30,31].

Based on the above reported simulation and the tests performed by the supplier to establish the MIC of each of the two new products, three different dosages of the P1 and P2 formulations were tested to evaluate their impact on the performance of *S. cerevisiae* during alcoholic fermentation (Table 1). Instead, only one dosage, based on the supplier’s recommendation, was used for P3 and P4 products (Table 1).

According to the European law (https://food.ec.europa.eu/plants/pesticides/maximum-residue-levels_en; accessed on 27 October 2024), specific Maximum Residues Limits (MRLs) have to be reported for synthetic products, such as P3. On the contrary, natural phytosanitary products, such as P1, P2, and P4, have no MRLs on the final product.

### 2.2. Fermentations Trials

A commercial strain of *S. cerevisiae*, Lalvin-EC1118 (Lallemand Inc., Montreal, QC, Canada) and a synthetic grape juice medium were used for the fermentation trials. The synthetic medium, prepared according to the OIV-OENO Resolution No. 370 [32] (2012; Table S1), was added with each phytosanitary product (P1–P4) at different dosages, calculated according to theoretical pesticide residuals (Table 1).

Finally, the medium was sterilized by filtration with a 0.45 μm of cellulose acetate filter. Fermentations were carried out in triplicate at 25 ± 2 °C in 300 mL Erlenmeyer flasks, containing 250 mL of the synthetic grape juice. The flasks were inoculated with a 48 h preculture, grown in the same synthetic medium, with cell concentrations of ~2 × 10^6^ cells/mL, determined by counting under microscopy light. Flasks, equipped with valves allowing the CO_2_ to escape, were incubated under static conditions, and weighed every day until the end of the fermentation (at a constant weight for two consecutive days) to monitor the fermentation kinetics.

### 2.3. Analysis

#### 2.3.1. Biomass Determination

Samples were taken from each flask throughout the alcoholic fermentation process, to evaluate the viable cell counts. An aliquot (100 μL) of serial dilutions of each sample was plated onto YPD agar medium (10 g/L yeast extract; 20 g/L peptone; 20 g/L dextrose, 20 g/L agar). The plates were then incubated at a constant temperature of 25 ± 2 °C.

#### 2.3.2. Analytical Determinations of the Fermentation Products

At the end of the alcoholic fermentation process (reducing sugars below 2 g/L), the fermented synthetic grape juice was centrifuged (8000× *g*, 4 °C, 10 min) to separate the yeast cells. The pellet was discarded, and the supernatant, after filtration through 0.45 μm cellulose membranes, was sparkled with nitrogen and stored at 15 ± 2 °C, in 100 mL amber glass bottles, until further analyses were performed. All the analyses were performed in duplicate.

##### Chemical Standard Parameters

Residual sugars, ethanol, and organic acid content were determined by high performance liquid chromatography (HPLC) [33]. After appropriate dilution in water, 20 μL of each sample was injected into the HPLC apparatus (Varian Inc., Palo Alto, CA, USA) equipped with a 410 series autosampler, a 210 series pump, and a 356-LC Refractive Index detector. Isocratic separation was performed at 75 °C on a Rezex-ROAOrganic Acids (30 + 15) cm × 7.8 mm i.d. column (Phenomenex, Torrance, CA, USA). The mobile phase was 10.5 mM H_2_SO_4_ at a flow rate of 0.6 mL/min. Each compound was quantified by comparison with its own external calibration curve (from 0.5 to 20 g/L), and the areas of the related peaks were recorded and integrated using Galaxie Chromatography Data System version 1.9.302.530 (Varian Inc., CA, USA).

##### Volatile Compounds

At the end of the alcoholic fermentation process, higher alcohols and acetoin were determined with a method previously developed [34] and using an AutoSystem XL gas chromatograph equipped with a flame ionization detector (FID) (Perkin Elmer, Shelton, CT, USA). A packed column (2 m × 2 mm o.d. tubing), packed with 80/100 mesh Carbopack C coated with 0.2% (*w*/*w*) Carbowax 1500, a product of Supelco (Sigma Aldrich, St. Louis, MO, USA), was used for all analyses. These volatile compounds were expressed as mg/L using a calibration curve obtained by the injection of the reference compounds (≥99% purity) purchased from Sigma (Sigma-Aldrich, Saint Louise, MO, USA). All samples were analyzed in triplicate. The free volatiles profile was determined by the HS-SPME GC-MS [35]. In detail, an AutoSystem XL gas chromatograph (Perkin Elmer) paired with a Turbomass Gold mass selective detector (Perkin Elmer) was used. The software used was TurboMass v.5.1.0. An Innowax column (30 m × 0.25 mm o.d., 0.25 μm film thickness, Agilent Technology, Santa Clara, CA, USA) was used. Volatile compounds were identified and quantitated by using the reference compounds (≥99% purity) purchased from Sigma (Sigma-Aldrich, Saint Louise, MO, USA). Compounds with no available reference standards were quantified based on the relative response to the 2-octanol internal standard. All samples were analyzed in duplicate.

##### Residual Volatile Compounds in the Phytosanitary Products

Volatile compounds present in the phytosanitary products were determined by using the same GC-MS apparatus and method as described above. Stock solutions for each phytosanitary product were prepared using a model wine solution (0.4% L (+)-tartaric acid, ethanol 12.5% *v*/*v*, pH 3.5). The composition of phytosanitary product solutions was compared with the composition of the fermented products to determine the residuals.

#### 2.3.3. Sensory Evaluation: Discriminant Test

A paired difference test [36] was applied to evaluate any sensory differences in the synthetic wines. Samples were evaluated for their olfactory characteristics by 9 expert judges. Samples, with three-digit numeric codes, were submitted anonymously in series with increasing concentrations. The instructions indicate to first examine the control (CTR), and then the samples, one pair at a time, with the following presentation sequence: P1A; P2A; P3; P4; CTR; P1B; P2B; P1C; P2C (see Table 1 for codes). The control (CTR) wine was inserted in order to verify the reliability of the provided answers. For each comparison, the judge had to answer whether, in his opinion, the samples examined were the same or different.

### 2.4. Statistical Analysis

Data were subjected to multivariate analysis of variance (MANOVA), using the statistical software Statgraphics Centurion (Ver. XV, StatPoint Technologies, Warrenton, VA, USA). The method used to discriminate pairwise differences between means uses Fisher’s LSD (Least Significant Difference) procedure; with this method, the risk of identifying each pair of statistically significant means when the difference is zero is 5%. There is significance when the *p*-value is <0.05. For the statistical analysis of the synthetic wine profiles, the variability factors were as follows: dose (A, B, C), phytosanitary products (P1, P2, P3, P4, CTR), fermentation replicates (a, b, c), and analysis replicates (1, 2, 3). All data and graphs relating to fermentation kinetics and the growth curve were processed with the program Excel^®^ from Microsoft^®^ 365 MSO (Ver. 2403 Build 16.0.17425.20176) 64 bit.

## 3. Results and Discussion

### 3.1. Fermentation Performance

**Figure 1:**
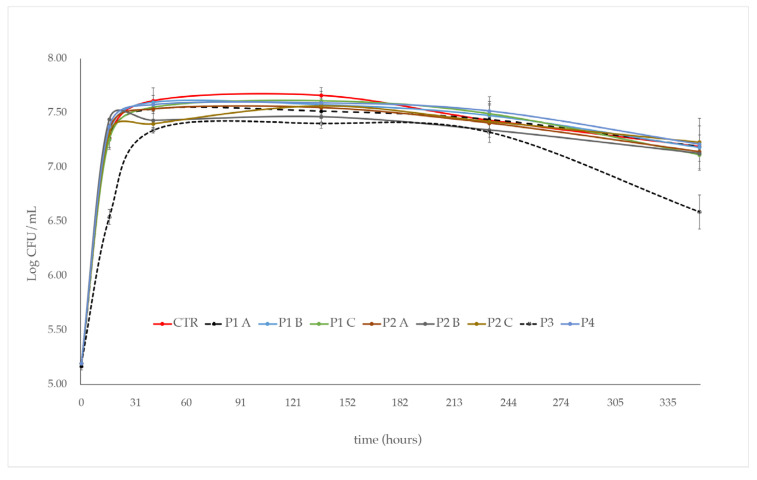
Growth kinetic of *S. cerevisiae* in the synthetic grape juice with the phytosanitary products added: P1 and P2 at three increasing doses (A, B, C); P3 and P4 at a single dose. Synthetic grape juice, without product addition, was used as control (CTR). Error bars represent standard deviation of three independent experiments.

The addition of P1 and P2 phytosanitary products did not affect the growth of *S. cerevisiae* during the first 16 h of alcoholic fermentation. Indeed, in both experimental trials, *S. cerevisiae* showed a biomass production comparable to that of the control (CTR) (2 × 10^7^ CFU/mL), regardless of the dose (A, B, C) of the product used (Figure 1). However, in comparison to the control (CTR), after a further 25 h of alcoholic fermentation, a slight dose-dependent decrease in *S. cerevisiae* growth was observed in the P2 treated samples. In any case, after two weeks of alcoholic fermentation, in the P1 and P2 treated samples, no matter the dose used, a cell concentration comparable to that of the control (~1.5 × 10^7^ CFU/mL), was observed (Figure 1).

The commercial product “3LOGY” (P4), used for comparative determination at the single dose of 0.04 mL/L, determined a growth kinetic of *S. cerevisiae* similar to that observed in the control (CTR). In contrast, the product “Switch” (P3), used at the concentration of 0.008 g/L, strongly affected the growth of *S. cerevisiae*. In comparison with the control, in the trials added with P3, a lower cell concentration was observed after the first 30 h of alcoholic fermentation. This low cell concentration was maintained throughout the alcoholic fermentation process until day ten; after that, a further significant growth decrease (~0.6 log units) was observed (Figure 1).

**Figure 2:**
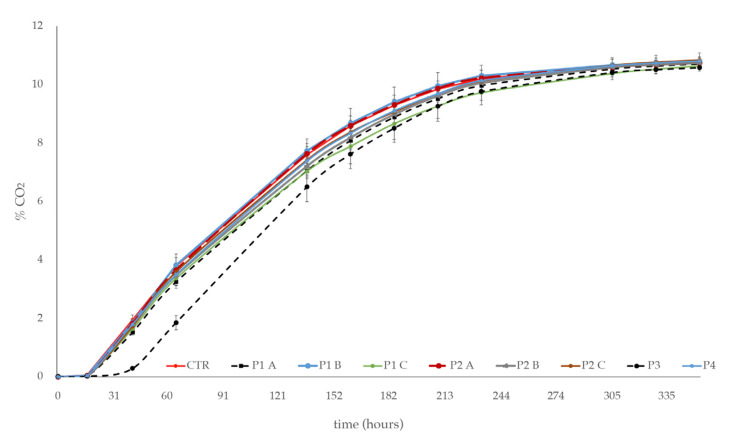
Fermentation kinetics of *S. cerevisiae* in the synthetic grape juice, with phytosanitary products added: P1 and P2, at three increasing doses (A, B, C); P3 and P4, at a single dose. Synthetic grape juice, without product addition, was used as control (CTR). Error bars represent standard deviations of three independent experiments.

The growth kinetics were in agreement with the fermentation kinetics (Figure 2). In particular, the fermentation rates of *S. cerevisiae* in the synthetic grape juice, with P1, P2, and P4 products added, irrespective to the dose used, were comparable to those observed in the CTR. Minor decreases in the P1 and P2 treated samples were observed. On the contrary, the P3 product was determined to have a delay of ~15 h for the start of the fermentation and a low fermentation rate through to the end of it. In any case, after 15 days, all the alcoholic fermentations were completed (residual sugar < 2 g/L).

Based on their origin (natural: P1, P2, and P4; and chemical: P3), phytosanitary treatment residuals at different concentrations in the synthetic grape juice showed a different impact on the fermentation and growth kinetics of *S. cerevisiae*. Residues of P1, P2, and P4 products have shown fermentation and growth kinetics similar to the control (CTR—no treated sample), regardless of the concentration used. On the other hand, the synthetic product (P3) negatively affected the growth of *S. cerevisiae* as well as the start and the progression of alcoholic fermentation. The impact of synthetic products on the yeast growth and metabolic activity have been reported by other authors [9,12,13,14,15,16,17,18,19,20,21,22] and different mechanisms of action have been hypothesized.

### 3.2. Chemical Composition of the Fermentation Products

#### 3.2.1. Chemical Standard Parameters

At the end of the alcoholic fermentation process, the main analytical parameters (organic acids, alcohols, and residual sugars) of the treated samples with each phytosanitary product and the control were analyzed (Table 2).

No statistical differences were detected in the fermented products for organic acids and residual sugar content. On the contrary, the P3 treated sample showed a significantly higher concentration (*p*-value = 0.0002) of glycerol (10.31 g/L) in comparison with that found in all the other samples, ranging from 7.61 and 8.42 g/L. It is worth highlighting that glycerol production by yeast is influenced by several environmental and growth factors [37,38].

Slight but significant differences in ethanol concentration, were also found (*p*-value < 0.05). In particular, the P3-treated sample showed a lower ethanol concentration in comparison with the samples treated with all the other phytosanitary products.

#### 3.2.2. Volatile Profile of the Fermentation Products

In general, samples treated with the phytosanitary products showed a significant increase (*p*-value < 0.005) in total fatty acid content, and a similar or lower content of total esters. Interestingly, the sum of total fatty acids increased in P1- and P2-treated samples in a dose dependent fashion. This result might be related to a higher production of fatty acids by *S. cerevisiae* in the presence of toxic molecules in the fermentation medium. Regarding the content of higher alcohols, such as n-propanol, 2-methyl-1-propanol, 2-methyl-1-butanol, and 3-methyl-1-butanol, no significant differences among the treated samples were observed. Noteworthily, all samples remained at concentrations lower than 350 mg/L, a threshold below which the higher alcohols do not negatively affect the quality characteristics of the wines [32]. Acetoin, an organic compound naturally produced by yeasts during alcoholic fermentation, was the only ketone detected, and it was significantly higher (*p*-value = 0.005) (12.367 mg/L) in the P3 treated sample in comparison with the control (CTR) (7.109 mg/L) and all the other fermented products (Table 3). Acetoin is considered a constituent of wine, and its presence is significant primarily in relation to the organoleptic properties of the wines [37]. It is formed during fermentation by the microbial activity of yeasts. In general, it is assumed that the Saccharomyces do not produce significant amounts of acetoin by the end of fermentation. In fact, it is produced in the early phase of fermentation, reaching its maximum of 25 to 100 mg/L at about the midway point, and then its content declines rapidly in the final stage of the process, presumably as a result of reduction to 2,3-butanediol. Consequently, normal dry wines fermented by *S. cerevisiae* generally contain acetoin, but at low levels [38].

At the end of the alcoholic fermentation process, samples treated with P1, P2, and P4 showed the presence of different amounts of residual volatile. It is worth mentioning here that the major compounds present in the EOs of the Apiaceae family (in P1 products) are α-pinene, limonene, carvone, linalool, thymol, menthol, (E)-anethole, and carvacrol [39]; those present in the EOs of the Lamiaceae family (in P1 and P2 products) are β-caryophyllene, linalool, limonene, β-pinene, 1,8-cineole, carvacrol, α-pinene, p-cymene, γ-terpinene, and thymol [40]; and those in EOs of the Geraniaceae family (in P2 products) are citronellol, geraniol, δ-elemene, δ-cadinene, α-ylangene, caryophyllene, humulene, β-selinene, and α-cedrol [41]. Some of these compounds (reported in Table 3 under “Total phytosanitary residual volatile compounds”) were found in the corresponding phytosanitary product (Table 3). The sample treated with P4 (commercial product “3LOGY”), showed the presence of eugenol (106.079 μg/L), geraniol (139.334 μg/L), and thymol (1347.985 μg/L), in agreement with the ingredients reported on the product label. Interestingly, residual volatile compounds of P1 and P2 phytosanitary products increase in the corresponding treated sample in a dose dependent fashion. As expected, no phytosanitary residues belonging to those investigated above were found in samples treated with P3. The results highlighted that the phytosanitary product residues showed a different impact on the volatile compounds of the fermented samples, according to the type of product and concentrations used. P1 and P2 treated samples showed the highest concentration of fatty acids, in a dose-dependent fashion. These natural phytosanitary products were characterized by the presence of EOs that are known to have lipophilic action and to act on the cell membranes of the fungus [28].

### 3.3. Sensory Analysis of Synthetic Wines

**Figure 3:**
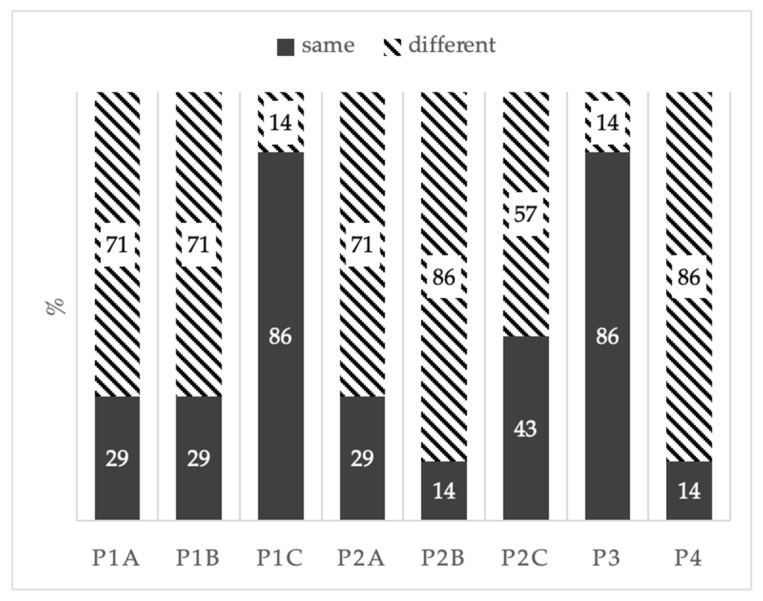
Discriminant difference test results of treated samples (P1, P2, P3, P4) versus untreated samples (CTR): A—lowest dose; B—intermediate dose; C—highest dose. Black bars: sample is different than the control (CTR); striped bars: sample is the same as the control (CTR).

A discriminant difference test (only for olfactory characteristics) was performed to verify the presence of any perceivable differences among the synthetic wines and the control (CTR). The responses showed significant differences among the tested samples (Figure 3). In particular, the P1 sample was distinguished from the control (CTR), although no clear dose correlation was observed. In fact, at the lowest (A) and intermediate (B) doses, six out of nine experts judges perceived a difference among the samples. Meanwhile, at the highest dose (C) only one response out of nine defined it as different. This could partially be explained by the adaptability of the sense of smell, but was still indicative of a small difference among the analyzed samples. Concerning the P2 samples, at the lowest and intermediate doses, six and eight out of nine expert judges, respectively, perceived a difference among P2A and P2B samples and the control, while at the highest dose only five out of nine defined it as different.

The P3 treated sample was indistinguishable, with three out of nine expert judges defining this sample as different. This is consistent with its known odorless characteristic. Samples treated with P4 showed an odor comparable to the two new formulation products (P1 and P2). However, seven out nine expert judges defined it as different compared to the control. All judges correctly recognized the control sample.

Sensory analysis highlighted differences between untreated and treated samples with the natural products (P1, P2 and P4). This could be related to the interaction of the phytosanitary product with the yeast metabolism or to the intrinsic odorous characteristic of the product itself. It is important to highlight that these results are obtained in synthetic grape juice and trials on real vineyard treatments should be carried out to validate these results. On the contrary, no olfactory differences were perceived by the experts for samples treated with the synthetic product (P3), consistent with its known odorless formulation.

## 4. Conclusions

Despite the presence of laws requiring compliance with waiting times for treatments, consumers are increasingly concerned about possible residues in grape juice, and, in turn, in the corresponding wine. To address this issue, research is increasingly oriented towards the use of plant protection products of natural origin. In this context, two new formulations of natural origin are being developed as phytosanitary products for vineyard treatments in the pre-harvest period.

The results of this study highlighted that both of the new natural EO-based phytosanitary products showed no significant impact on the growth and on the fermentation kinetics of the commercial yeast strain *S. cerevisiae* EC1118, at any of the tested concentrations. Therefore, these products represent interesting alternative tools applicable in organic farming and in the pre-harvest period.

Further studies need to evaluate the application of these new phytosanitary products in vineyards and to monitor the actual residuals of the product on the corresponding grape juice. In addition, any possible correlation/effect on the wine’s attributes derived from different phytosanitary products needs to be analyzed based on the varietal characteristics of the grapes.

## Figures and Tables

**Table 1:** Phytosanitary products and residual doses added in the synthetic grape juice.

Product	Code	Manufacturer Company	MRLs ^1^ (mg/kg)	Recommended Dose (mL/hL)	Residual Dose in Synthetic Grape Juice (mL/L)
P1	P1A	Institute for Environmental Solutions		270	0.027
	P1B		No limit	330	0.033
	P1C			390	0.039
P2	P2A	Institute for Environmental Solutions		200	0.020
	P2B		No limit	400	0.040
	P2C			800	0.080
P3	P3	Syngenta	Limit	80 ^2^	0.008 ^3^
P4	P4	Sipcam Italia	No limit	400	0.040
CTR	CTR	-	-	-	-

^1^ MRLs: Maximum Residues Limits: MRL is 0.02 mg/kg for cyprodinil; MRL is 10 mg/kg for fludioxonil; ^2^ g/hL; ^3^ g/L.

**Table 2:** Organic acids, alcohols, and residual sugar content at the end of the alcoholic fermentation of synthetic grape juice samples treated with phytosanitary products. Data are representative of three independent experiments and expressed as the mean of two analytical determinations. Within each column, values followed by the same letters (a–d) are not statistically significantly different.

Sample	Ethanol ^1^	Glycerol ^2^	Tartaric Acid ^2^	Citric Acid ^2^	Succinic Acid ^2^	Fructose ^2^	Glucose ^2^
P1A	15.90 abc	7.95 abc	1.24 a	0.50 a	1.00 ab	0.84 a	nd
P1B	16.33 c	8.31 bc	1.13 a	0.54 ab	0.63 a	1.48 a	nd
P1C	15.57 abc	7.62 a	1.20 a	0.52 ab	0.86 ab	2.27 a	nd
P2A	16.28 c	8.42 c	1.15 a	0.69 c	1.12 ab	0.90 a	nd
P2B	16.02 bc	7.81 abc	1.19 a	0.51 ab	0.78 ab	1.19 a	nd
P2C	15.13 a	7.74 ab	1.17 a	0.58 abc	0.97 ab	1.65 a	nd
P3	15.19 a	10.31 d	1.10 a	0.56 a	1.07 ab	2.27 a	nd
P4	15.27 ab	7.95 abc	1.10 a	0.55 ab	1.00 ab	0.85 a	nd
CTR	15.84 abc	8.23 abc	1.20 a	0.64 bc	1.25 b	1.10 a	nd

^1^ expressed as % *v*/*v*; ^2^ expressed as g/L; nd: not detected.

**Table 3:** Volatile profiles of the fermented products.

Compound	CTR	P1A	P1B	P1C	P2A	P2B	P2C	P3	P4	*p*-Value
isoamyl acetate ^1^	57.326 bc	27.774 a	42.071 abc	34.647 ab	36.247 ab	31.035 a	47.105 abc	60.649 c	36.552 ab	ns
ethyl caproate ^1^	81.024 cd	77.605 cd	65.956 ab	73.667 bc	79.384 cd	79.082 cd	83.295 d	62.045 a	72.983 bc	0.005
ethyl acetate ^5^	29.197 ab	26.439 ab	28.316 ab	25.658 a	29.928 ab	26.699 ab	28.865 ab	31.465 ab	34.157 b	ns
ethyl octanonate ^1^	120.292 c	133.609 d	75.591 a	119.702 c	75.474 a	109.384 bc	100.016 b	100.357 b	109.777 bc	0.000
ethyl decanoate ^2^	69.893 bc	75.251 bcde	71.676 bcd	90.241 e	38.897 a	85.300 cde	80.682 bcde	80.682 de	65.414 b	0.002
ethyl undecanoate ^2^	5.074 b	5.991 c	6.336 c	12.786 e	4.115 a	4.991 b	5.101 b	6.212 c	8.353 d	0.000
isoamyl octanoate ^3^	9.538 c	6.668 abc	6.082 ab	7.795 abc	4.917 a	8.894 bc	9.277 bc	14.298 d	8.756 bc	0.007
diethylsuccinate ^1^	48.882 b	40.628 a	48.966 b	49.564 b	39.543 a	44.014 ab	43.728 ab	46.423 ab	45.862 ab	ns
**Total esters ^1^**	**29,588.550 ab**	**26,806.745 a**	**28,632.570 ab**	**26,046.572 a**	**30,206.832 abc**	**27,061.919 a**	**29,163.173 ab**	**31,836.034 bc**	**34,503.872 c**	**0.023**
octanoic acid ^4^	34.991 ab	40.763 bc	38.371 bc	48.968 d	29.458 a	50.230 d	49.268 d	42.896 c	43.103 c	0.000
nonanoic acid ^4^	20.872 ab	24.007 bc	23.977 bc	37.252 e	15.138 a	29.612 cd	32.863 de	36.006 e	25.100 bc	0.000
decanoic acid ^4^	1.373 b	nd	nd	nd	nd	nd	nd	2.093 c	2.469 d	0.000
**Total fatty acids ^4^**	**57.237 b**	**64.769 c**	**62.348 bc**	**86.220 f**	**44.596 a**	**79.841 e**	**82.131 ef**	**80.994 ef**	**70.671 d**	**0.000**
1-propanol ^5^	27.260 ab	23.995 a	30.662 ab	22.830 a	28.032 ab	25.003 ab	26.275 ab	32.819 b	26.355 ab	ns
2-methyl-1-propanol ^5^	35.737 ab	29.141 ab	37.386 b	26.758 a	37.935 b	29.295 ab	28.919 ab	33.093 ab	29.482 ab	ns
2-methyl-1-butanol ^5^	22.511 abc	20.052 ab	22.670 bc	19.222 a	22.971 bc	19.731 ab	20.199 ab	23.829 c	21.398 abc	ns
3-methyl-1-butanol ^5^	74.262 b	59.444 ab	75.016 b	54.272 a	76.836 b	64.968 ab	63.822 ab	67.494 ab	66.511 ab	ns
1-octanol ^5^	0.001 c	0.002 f	0.002 d	0.002 e	0.001 c	0.001 c	0.001 b	0.001 a	0.001 b	0.0000
**Total aliphatic alcohols ^5^**	**159.771 b**	**132.633 ab**	**165.736 b**	**123.085 a**	**165.775 b**	**138.998 ab**	**139.215 ab**	**157.236 ab**	**143.748 ab**	**ns**
2-phenylethanol ^5^	33.650 e	27.556 a	30.817 cd	30.822 cd	27.891 ab	31.562 cde	33.485 e	30.065 bc	33.164 de	0.0003
**Total aromatic alcohols ^5^**	**33.650 e**	**27.556 a**	**30.817 cd**	**30.822 cd**	**27.891 ab**	**31.562 cde**	**33.485 e**	**30.065 bc**	**33.164 de**	**0.0003**
Acetoin ^5^	7.109 a	8.494 ab	8.632 ab	9.540 b	8.472 ab	8.346 ab	8.879 ab	12.367 c	8.574 ab	0.005
**Total ketones ^5^**	**7.109 a**	**8.494 ab**	**8.632 ab**	**9.540 b**	**8.472 ab**	**8.346 ab**	**8.879 ab**	**12.367 c**	**8.574 ab**	**0.005**
**Total phytosanitary product residual (essential oils) ^1^**	**nd**	**1637.037 b**	**1803.497 c**	**2675.900 e**	**643.082 a**	**1906.286 d**	**3485.375 f**	**nd**	**1593.398 b**	

All data are expressed as the average of 3 replicates. Within each row, values followed by the same letters (a–f) are not statistically significantly different. ^1^ expressed as μg/L; ^2^ expressed as μg/L of ethyl octanoate; ^3^ expressed as μg/L of isoamyl acetate; ^4^ expressed as μg/L of 2-octanol; ^5^ expressed as mg/L; ns: not significant; nd: not detected.

## Data Availability

The original contributions presented in the study are included in the article and Supplementary Materials, further inquiries can be directed to the corresponding author.

## Supplementary Materials

The following supporting information can be downloaded at: https://www.mdpi.com/article/10.3390/foods13213484/s1, Table S1: Composition of the chemically-defined grape juice medium reported by Henschke and Jiranek (1993) containing amino acid quantity to give a nitrogen concentration of 200 mg/L, and a sugar amount (230 g/L).
