# Supplementary material for: Impact of Natural Phytosanitary Product Residuals on Yeast Fermentation Performance and Wine Composition
Source: Foods. 2024 Oct 30;13(21):3484. doi: 10.3390/foods13213484 (PMC11545603; doi:10.3390/foods13213484)
Supplement: Supplementary file 1 [file foods-13-03484-s001.zip › foods-3243182-supplementary.pdf]

Supplemental Tables

Table S1. Composition of the chemically-defined grape juice medium reported by Henschke and Jiranek (1993) containing amino acid quantity to give a nitrogen concentration of 200 mg/L, and a sugar amount (230 g/L).

	Component	Amount per litre
**Carbon source**	Glucose	115 g
	Fructose	115 g
**Organic acid**	Potassium tartrate	5.00 g
	Citric acid	0.20 g
	L-malic acid	3.00 g
**Salts**	K_2HPO_4	1.14 g
	$MgSO_4{\cdot}7H_2O$	1.23 g
	$CaCl_2{\cdot}2H_2O$	0.44 g
	$MnCl_2{\cdot}4H_2O$	198.2 μg
	$ZnCl_2$	135.5 μg
	$CuCl_2$	13.6 μg
	$FeCl_2$	32.0 μg
	H_3BO_3	5.7 μg
	$Co(NO)_3{\cdot}6H_2O$	29.1 μg
	$NaMoO_4{\cdot}2H_2O$	24.2 μg
	KIO_3	10.8 μg
**Vitamins**	Myo-inositol	20 mg
	Pyridoxine·hydrocl oride	0.40 mg
	Nicotinic acid	0.40 mg
	Calcium pantotenate	0.20 mg
	Thiamine hydrocloride	0.10 mg
	p-Aminobenzoic acid	0.04 mg
	Riboflavin	0.04 mg
	Biotin	0.03 mg
	Folic acid	0.04 mg
**Amino Acids**	Aspartic acid	89 mg
	Glutamic acid	126 mg
	Alanine	26 mg
	Arginine	188 mg
	Asparagine	39 mg
	Phenylalanine	39 mg
	Glycine	14 mg
	Glutamine	51 mg
	Isoleucine	51 mg
	Histidine	39 mg
	Leucine	76 mg
	Lysine	63 mg
	Methionine	39 mg
	Proline	126 mg
	Serine	101 mg
	Tyrosine	6.00 mg
	Threonine	89 mg
	Tryptophan	26 mg

	Valine	51 mg
**Nitrogen sources**	$(NH_4)_2HPO_4$	100 mg
**Lipids**	Ergosterol	10 mg
	Tween 80®	0.5 mL
**pH**		3.2 - 3.5
